# Tracking cell layer contribution during repair of the tympanic membrane

**DOI:** 10.1242/dmm.050466

**Published:** 2024-03-28

**Authors:** Olivia M. Dinwoodie, Abigail S. Tucker, Juan M. Fons

**Affiliations:** Centre for Craniofacial and Regenerative Biology, Faculty of Dentistry, Oral and Craniofacial Sciences, King's College London, London SE1 9RT, UK

**Keywords:** Eardrum, Proliferation, Wound healing, Germ layer

## Abstract

The tympanic membrane (i.e. eardrum) sits at the interface between the middle and external ear. The tympanic membrane is composed of three layers: an outer ectoderm-derived layer, a middle neural crest-derived fibroblast layer with contribution from the mesoderm-derived vasculature, and an inner endoderm-derived mucosal layer. These layers form a thin sandwich that is often perforated following trauma, pressure changes or middle ear inflammation. During healing, cells need to bridge the perforation in the absence of an initial scaffold. Here, we assessed the contribution, timing and interaction of the different layers during membrane repair by using markers and reporter mice. We showed that the ectodermal layer is retracted after perforation, before proliferating away from the wound edge, with keratin 5 basal cells migrating over the hole to bridge the gap. The mesenchymal and mucosal layers then used this scaffold to complete the repair, followed by advancement of the vasculature. Finally, differentiation of the epithelium led to formation of a scab. Our results reveal the dynamics and interconnections between the embryonic germ layers during repair and highlight how defects might occur.

## Teaser

**Editor's choice:** This paper follows the wound healing process after perforation of the tympanic membrane (i.e. eardrum) in transgenic mouse models, focusing on the contribution of different germ layers and highlighting how defects might arise.

## INTRODUCTION

**Fig. 1.**
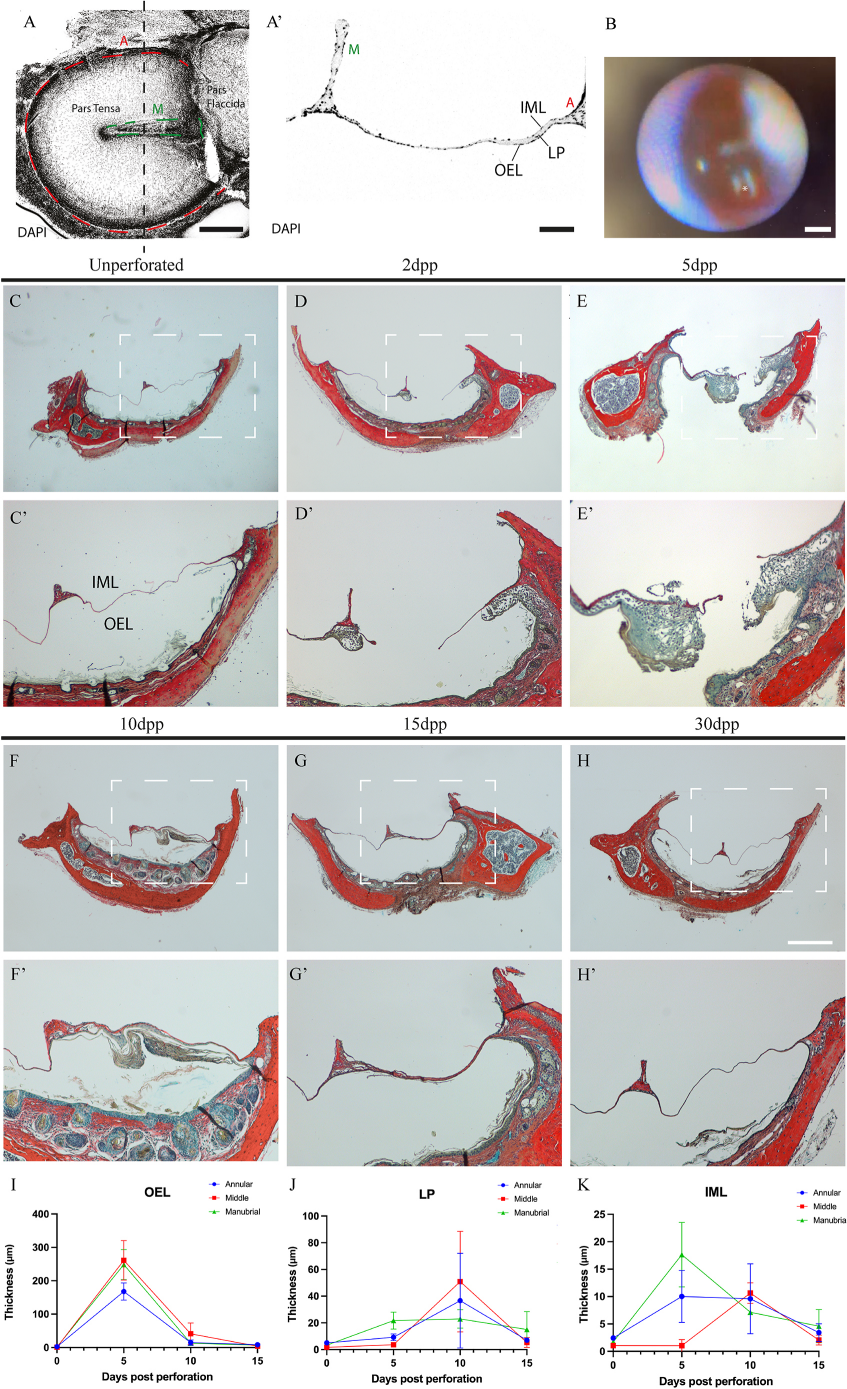
**The tympanic membrane heals within 30 days post perforation.** (A,A′) Whole-mount view (A) of a dissected tympanic membrane (TM) imaged from the middle ear side, showing pars tensa and pars flaccida of the TM, the manubrium (M, indicated by the green lines) of the malleus and the annulus tympanicus (A, red dashed line). Nuclei (DAPI) are stained with DAPI (grey). A transversal section (A′) at the level indicated by the vertical dashed line is shown in A, highlighting the manubrium (M, green), the inner mucosal layer (IML), the lamina propria (LP), the outer epithelial layer (OEL) and the annulus tympanicus (A, red). Scale bars: 500 µm (A), 100 µm (A′). (B) Otoscopy photograph of a perforated TM. The asterisk (*) marks the site of perforation. Scale bar: 500 µm. (C-H′) Trichrome staining of the TM, showing the unperforated TM (C,C’) and its healing process after perforation at 2, 5, 10, 15 and 30 days post perforation (dpp) (D-H′). Boxed areas in C-H are shown magnified in C′-H′. Scale bar: 500 µm. (I-K) Quantification of the thickness of the three TM layers, i.e. the OEL, the LP and the IML during repair. Plotted is the thickness (in μm) of annulus tympanicus (Annular, blue), manubrium (Manubrial, green) and malleus (Middle, red) at 0, 5, 10 and 15 dpp. Error bars indicate the ±s.d. WT mice were used for histology, *n*>10 for each stage. For quantification *n*=4 mice were used per timepoint.

The tympanic membrane (TM) is located between the external and the middle ear, where sound vibrations are picked up and transmitted via the manubrial insertion at the centre of the TM through the ossicular chain and to the cochlea. The TM has two main parts, the pars tensa (PT) which is the site of sound conduction, and the pars flaccida (PF) the role of which is thought to involve pressure equilibration (Lim, 1995; Robert et al., 1982) (Fig. 1A). The PT houses the annulus tympanicus; the thicker outer ring of the TM, as well as the manubrium of the malleus, which is where the TM connects to the ossicular chain of the middle ear. The PT is stretched taut, held by the tympanic ring, with the outer epithelial and inner mucosal layers (OEL and IML, respectively) continuous with the external ear canal and mucosa of the middle ear, respectively (Tucker et al., 2018).

The TM represents a unique example of a single tissue derived from all three germ layers (Takechi et al., 2016). The OEL, derived from the ectoderm, consists of keratinocytes. These vary according to their extent of differentiation and resemble a cellular hierarchy reminiscent of the cell types in the epidermis of the skin (Frumm et al., 2021). The underlying lamina propria consists of neural crest-derived mesenchyme, with contribution from the mesoderm-derived blood vessels and nerves of the TM, and can be identified via *Wnt1* lineage tracing (Chai et al., 2000). Finally, the IML is endoderm derived and can be identified by *Sox17* lineage tracing (Thompson and Tucker, 2013; Nowotschin et al., 2019). The lamina propria is thickened towards the annulus and manubrium and extremely thin towards the centre of the PT (Fig. 1A′). The PT and PF have a similar layered structure but differ in the make-up of their lamina proprias. In the PT, this layer contains highly assembled radial and circular collagen fibres, consisting mainly of type II collagen, whereas type I collagen is found more loosely arranged in the PF (Stenfeldt et al., 2006; Knutsson et al., 2007).

During development, ectodermal cells from the region of the first pharyngeal arch invaginate towards the endodermal out-pocketing of the first pharyngeal pouch (Minoux et al., 2013). The bones of the middle ear form from the neural crest-derived mesenchyme (Richter et al., 2010). As the ectodermally derived external acoustic meatus invaginates (Fons et al., 2020) and the endodermal middle ear cavity extends, the two layers enclose a layer of mesenchyme between them, ultimately resulting in the typical trilaminar structure of the TM (Mallo, 2001).

Acute eardrum perforation is a major clinical presentation and can result from pressure changes, trauma and infection. In 94% of cases, this perforation can heal itself with no scarring and no changes to original hearing capability. By contrast, 6% of cases do not heal and become chronic, posing a strain on patients and health services worldwide (Dolhi and Weimer, 2023).

Previous studies have revealed a general account of TM perforation healing in a range of mammalian species, including guineapig, cat, rat and mouse (Gladstone et al., 1995; Stenfors et al., 1980). Histological analysis in rat has suggested the potential timing of the reaction from each of the layers, with an initial epithelial response, followed by those from the middle and inner layers (de Araújo et al., 2014). In the cat, the perforation edge was found to thicken with an epithelial bridge observed 7-9 days after TM perforation (Stenfors et al., 1980). A retraction of the epithelium was noticed 48 h after perforation, leaving an uncovered middle layer (Stenfors et al., 1980). A similar retraction was observed in histological section in the rat, with collagen fibres left exposed (de Araújo et al., 2014). However, due to the thinness of the membrane, it is difficult to follow the contribution of the different layers without molecular markers.

In intact eardrums, proliferative centres have been noticed to form around the annulus and near the manubrium (Chari et al., 2019; Tucker et al., 2018). Following perforation, a large increase in the number of proliferating cells have been shown in all three layers, but highest in the ectoderm layer (Chari et al., 2019). Interestingly, similar to homeostasis, proliferation after perforation was focused to areas near the annulus and the malleus (Chari et al., 2019). Proliferation at a distance from the wound site has also been noticed in the guineapig, suggesting that epithelial cells migrate to the wound site from distinct regions (Clawson and Litton, 1971; Reeve, 1977). Ink-tracing experiments have suggested that the basal and spinosum layers contribute to repair (Gladstone et al., 1995), with expression of the basal marker keratin 5 (KRT5) upregulated during repair (Chari et al., 2019). If proliferation is inhibited by using Mitomycin C in rats, the perforations fail to heal even after 2 months, highlighting the essential role of proliferation (Güneri et al., 2003).

The phases of TM wound healing have been classified into stages by using microarray analysis, mirroring the phases noticed during skin wound healing (Santa Maria et al., 2011). Immediately following perforation is the inflammatory phase, whereby genes involved in acute inflammation are upregulated, as well as some growth factors (e.g. EGF) (Santa Maria et al., 2011). This phase takes place almost immediately following perforation to 2 dpp (days post perforation). Following the inflammatory phase, the proliferative phase (i.e. 2-5 dpp) initiates with upregulation of signalling pathways, such as FGFs and PDGF (Santa Maria et al., 2011). Finally, a remodelling phase occurs (7-14 dpp), whereby thinning of the tissue takes place (Santa Maria et al., 2011). It is worth noting that although TM healing is often compared to skin wound healing there are differences. Importantly, skin wound healing requires an initial scaffold of fibroblasts, creating a platform for re-epithelialization, while the TM appears to involve migration of keratinocytes to bridge the gap and to create an initial scaffold along which the fibroblasts migrate (Gladstone et al., 1995). Interestingly, one study labelling TM perforations with ink, suggested that the IML formed the initial bridge across the perforation along which the ectodermal cells then migrated (Johnson and Hawke, 1986). The order in which the layers migrate is, therefore, unclear. That the middle, fibroblast layer arises last is supported by studies in humans, during which the perforation was replaced by a bi-layered membrane without the fibroblast layer or by a membrane with disorganized fibrils (Yamashita, 1985).

While the focus of previous work has rested on the presence of potential stem cell populations and surgical options for enhancing repair, further investigation is required to understand the cellular contribution, precise timing and mechanisms of the healing process. In this study, we used transgenic reporter mouse models to show how cellular components from each of the three TM layers contribute to the repair of the tissue, uncovering novel parts of the healing process and highlighting the timing of reaction for each cell layer. We show that the epithelial layer initially retracted post perforation, leaving behind both the inner and middle layers. The retraction was followed by formation of the epithelial scaffold, which was composed of localized areas of proliferating KRT5-positive keratinocytes that later matured. We show that the mesenchymal and endodermal layers responded later, with proliferation and migration in tandem with changes to the vasculature. Understanding the mechanisms of repair in the TM is crucial as, without this knowledge, we cannot begin to design treatments to enhance repair in acute as well as chronic TM perforations.

## RESULTS

### Small tympanic membrane perforations resolve over 30 days in the mouse

To understand the timing of the healing process, repair was characterised in wild-type mice by using histological analysis from the day of perforation up to 30 dpp. Perforations were consistently placed in the right lower quadrant of the PT, between the manubrium and annulus, using an endoscopic camera to allow visualization during perforation (Fig. 1B). Mice were culled at different timepoints to create a time series. Compared with unperforated ears, a distinct hole was present 2 days after perforation, with a pronounced thickening of the membrane away from the injury near to the annulus and manubrium (Fig. 1C,D,C′,D′). Five days after injury, the membrane continued to thicken on the outside of the eardrum. At this stage ∼50% of ears analysed still showed a centre hole where the perforation had been made, whereas a bridge over the hole was evident in the other 50% (Fig. 1E,E′ and data not shown, *n*>10). By day 10, a bridge had formed over the injury site, with evidence of keratinisation and cell shedding into the external ear canal (Fig. 1F,F′). After 15 dpp, the membrane was almost back to normal thickness compared to the uninjured side of the membrane (Fig. 1G,G′); by 30 dpp no overt signs of any previous damage were present (Fig. 1H,H′). To quantify the thickness of the TM layers during the healing process, measurements of each layer were taken at three regions across the drum at three timepoints (Fig. S1.1) (*n*=4 per stage). The OEL reached its peak thickness at 5 dpp (Fig. 1I). By 10 dpp, the thickness of the OEL had reduced and was back to baseline by 15 dpp (Fig. 1I). The lamina propria followed a different trajectory, with peak thickness reached at 10 dpp, although the extent of thickening was far less than that of the OEL (Fig. 1J). The IML showed thickening at 5 dpp, which lasted until 10 dpp before returning to baseline at 15 dpp, illustrating a more immediate response than that of the lamina propria (Fig. 1K). However, while IML thickness did increase – from 1 μm up to ∼16 μm (Fig. 1K), the increase in OEL thickness was several magnitudes higher – from ∼1 μm to ∼270 μm (Fig. 1I).

### The outer epithelial layer retracts leaving behind the middle and inner mucosal layers

**Fig. 2.**
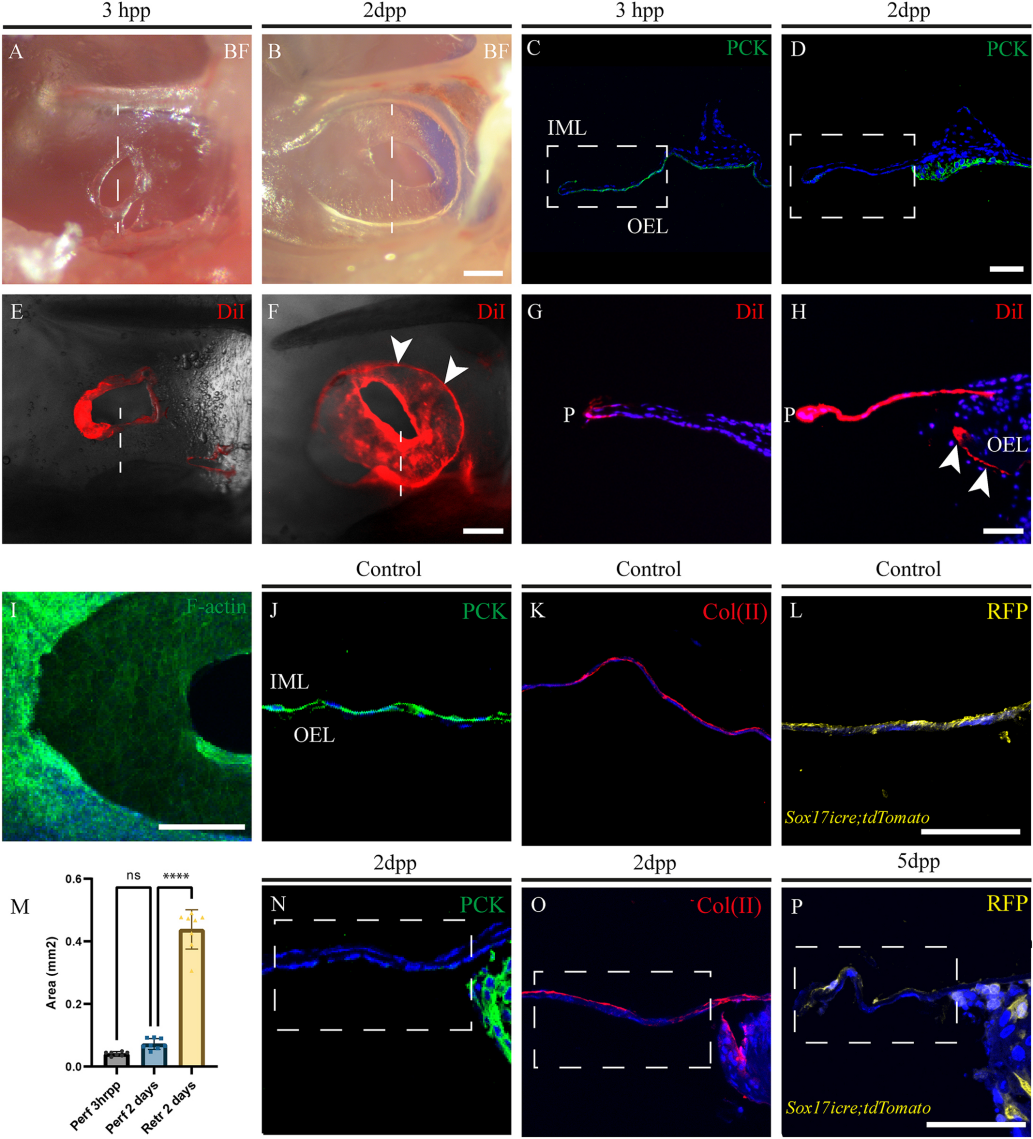
**Retraction of the outer epithelial layer post perforation of the tympanic membrane.** (A,B) Bright-field (BF) images of the tympanic membrane (TM) of wild-type mice 3 hours post perforation (hpp) and 2 days post perforation (dpp) as indicated. At 2 dpp a large retraction was observed compared to the 2 hpp timepoint. Scale bar: 200 µm. (C,D) Immunofluorescence images of transversal TM sections obtained at the level indicated by dashed vertical lines in A and B, respectively, stained with the epithelial marker pan-cytokeratin (PKC, green). Boxed areas indicate unretracted membrane. Scale bar: 50 µm. (E,F) Whole-mount images of TM perforation sites stained with DiI (red) at 3 hpp (E) and 2 dpp (F). Arrowheads point to areas of DiI-positive retracted membrane. Dashed vertical lines indicate the plane of section in panels G and H. Scale bar: 200 µm. (G,H) Immunofluorescence images of TM transversal sections stained with DiI at 3 hpp (G) and 2 dpp (H). Arrowheads denote DiI-stained cells in the retracted OEL. Scale bar: 50 µm. (I) Whole-mount fluorescence image of perforated TM area showing actin filaments stained with phalloidin (green). Scale bar: 100 µm. (J-L, N-P) Immunofluorescence images of the TM before (J-L) and after (N-P) perforation at the indicated timepoints. TMs from wild-type mice were stained for wide-spectrum cytokeratins using pan-cytokeratin (PCK, green; J,N) or for type II collagen [Col(II), red; K,O]. TMs from *Sox17icre;tdTomato* mice were stained with red fluorescent protein (RFP, yellow; L,P). Nuclei were stained with DAPI (blue). Boxed areas in N-P indicate unretracted membrane. Experimental replicates: 3 hpp (*n*=4), 2 dpp (*n*=4), 5 dpp (*n*=2). DiI staining experiments: *n*=3 per timepoint. Immunofluorescence assays were repeated three times on different specimens. IML, inner mucosal layer; P, perforation; OEL, outer epithelial layer. (M) Bar graph showing the area of TM perforation (in mm^2^) of wild-type mice at 3 hpp (grey) and 2 dpp (blue), and of the retracted region at 2 dpp (yellow), with *n*=7 (3 hpp), *n*=8 (2 dpp). Values are the mean±s.d., ****P<0.0001 (one-way ANOVA with Tukey's post hoc test). ns, not significant.

Histological analysis at 2 dpp suggested a retraction of tissue away from the initial wound site. A similar early retraction of tissue away from the perforation has previously been described in the rat and cat (Reeve, 1977; Stenfors et al., 1980), but has not been described in more-recent literature. In wholemount, no retraction was evident 3 h post perforation (hpp), but a large retraction was clearly evident 2 dpp (Fig. 2A,B). The fact that we observed no retraction of tissue away from the site of perforation after 3 h, suggests that this effect was not due to an immediate release of mechanical tension within the eardrum. To further analyse whether the cells had retracted or sloughed off, DiI dye was used to label cells directly around the perforated region. At 3 hpp, DiI staining was observed for cells around the perforation site (Fig. 2E). By 2 dpp, DiI-stained cells could be observed all around the retracted part of the membrane (Fig. 2F), highlighting that a true retraction had occurred. This was further analysed by observing the labelled cells in section. At 3 hpp, only cells around the perforated region were DiI positive (Fig. 2G) while, at 2 dpp, the staining could be seen within the OEL far from the perforation site, illustrating the fact that these cells had moved away from the injury site (arrowheads, Fig. 2H). Perforated TMs were stained with phalloidin, which labels F-actin cables, to outline the cells in the retracted and unretracted regions at 2 dpp (Fig. 2I). High phalloidin levels were evident around the retracted tissue but not in the cells left behind around the perforation site (Fig. 2I). The area of the perforation was measured compared to the area retracted (Fig. 2M) (*n*=7-8). The area of the actual perforation showed no significant change between 3 h and 2 days; however, at day 2, the membrane showed a very consistent area of retraction, ∼3-4 times the area of the original perforation (Fig. 2M).

In the cat and rat retraction has been suggested to be due to pulling away of the OEL. To confirm which layer had retracted, TMs were sectioned (Fig. 2A,B dotted lines) and stained for pan-cytokeratin, a marker of the OEL (Fig. 2J). At 3 hpp pan-cytokeratin-positive cells were observed in the outer layer reaching the edge of the perforation (Fig. 2C), while by 2 dpp the regions staining for pan-cytokeratin was limited to the retracted membrane (Fig. 2D,N). The outer ectoderm-derived epithelium, therefore, retracts as part of the early wound response. Having shown retraction of the OEL after perforation, the nature of the tissue that remained around the perforation was analysed. Collagen II was used as a marker of the lamina propria to discern whether the unretracted membrane was composed of middle layer tissue (Fig. 2K). In contrast to the loss of pan-cytokeratin (Fig. 2N), staining for collagen II was observed in the unretracted membrane (Fig. 2O), suggesting that the part of the membrane ‘left behind’ was the lamina propria. As the IML is of endodermal embryonic origin, a *Sox17icre;tdTomato* reporter mouse was used to identify endoderm-derived tissues in the adult at 5 dpp (Fig. 2L). RFP-positive cells, stained in yellow, were evident in the unretracted membrane (Fig. 2P), highlighting that, while the OEL retracted away from the wound site after injury, the mesenchymal and endodermal layers remained at the perforation site.

### Basal epithelial cells cover the perforation and create a scaffold

**Fig. 3.**
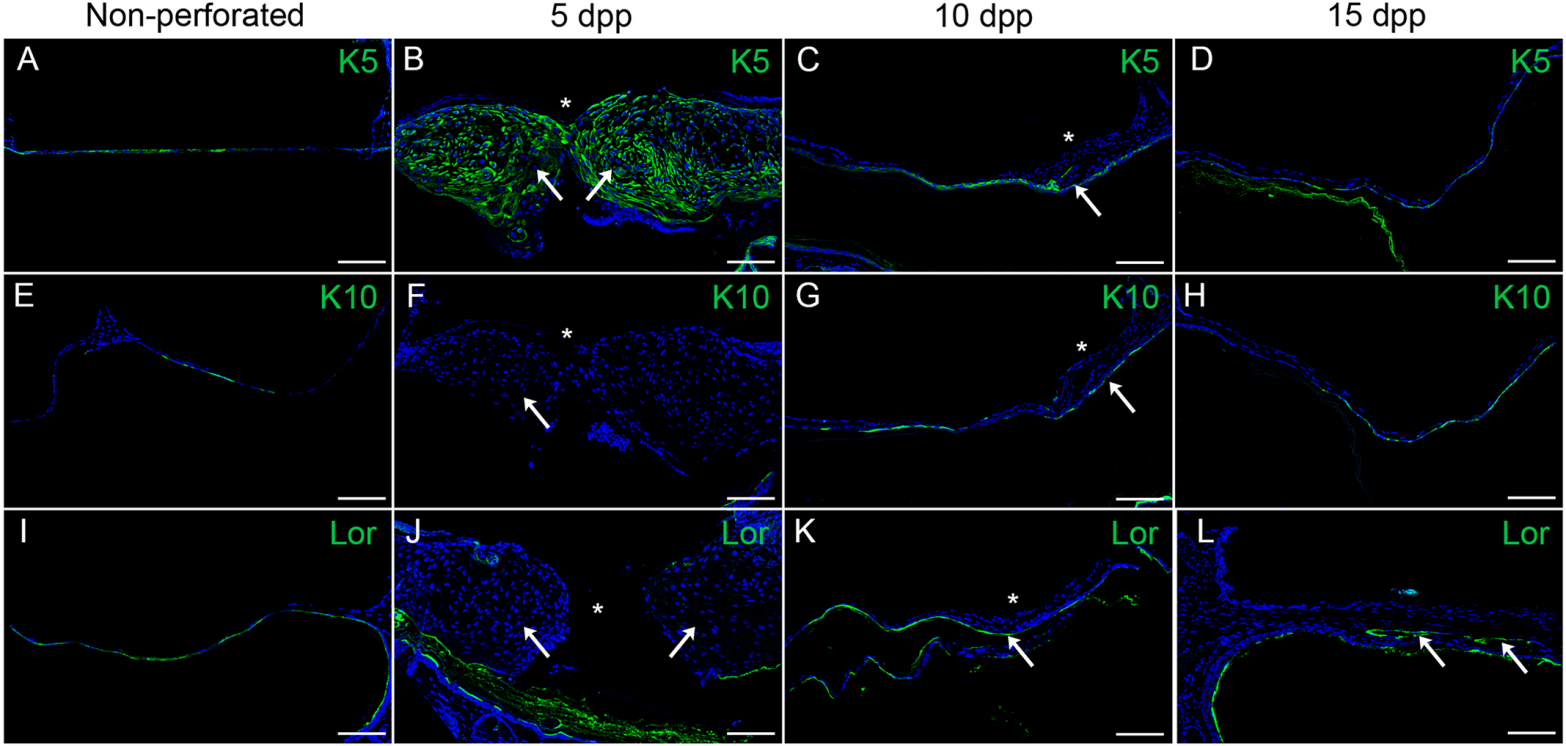
**Perforation closure of the tympanic membrane by undifferentiated basal cells followed by differentiation.** (A-L) Immunofluorescence images of wild-type mice showing the three epithelial layers of the tympanic membrane (TM) before perforation (Non-perforated) and at 5, 10 and 15 days post perforation (dpp) as indicated. Staining was for the basal cell marker keratin 5 (K5, green; A-D), the spinous layer marker keratin 10 (K10, green; E-H) and the cornified layer marker loricrin (Lor, green; I-L). Arrows in B,F and J indicate the presence of Krt5- positive cells and lack of Krt10- and loricrin-positive cells. Arrows in C,G, and K indicate a reduction of Krt5-positive cells and appearance of Krt10- and loricrin-positive cells at the perforation site. Arrows in K and L indicate the sites where peeling off occurs. Asterisks (*) indicate the perforation site. Nuclei were stained with DAPI (blue). Scale bars: 80 µm. *n*=3 per timepoint.

To investigate the contribution of the OEL to the healing of the perforated TM, three epithelial markers of specific stages of keratinocyte differentiation were analysed. Keratin 5 (KRT5) marks undifferentiated basal epithelial stem cells, while keratin 10 (KRT10) and loricrin mark committed and terminally differentiated keratinocytes, respectively (Koster et al., 2004). The unperforated eardrum has been shown to have a three-dimensional differentiation hierarchy of keratinocytes, with clusters of undifferentiated and differentiated cells (Frumm et al., 2021). In keeping with this, in sections through unperforated TMs, KRT5-, KRT10- and loricrin-positive cells were present within the ectodermal layer (Fig. 3A,E,I). Between 5 and 7 dpp, the epithelium had expanded and created a scaffolding covering the perforated site (Fig. 3B). The newly constructed bridge was KRT5 positive (Fig. 3B) but, unlike the eardrum during homeostasis, contained no KRT10- and loricrin-positive cells (Fig. 3F,J). Healing of the wound therefore involves the undifferentiated, basal cell population. By 10 dpp a pronounced reduction of KRT5-positive cells was observed (Fig. 3C) as well as the appearance of committed (KRT10-positive) keratinocytes (Fig. 3G) and terminally differentiated (loricrin-positive) keratinocytes (Fig. 3K), indicating that the cells involved in the initial epithelial ingrowth had started to differentiate. The presence of loricrin-positive keratinocytes at days 10 and 15 post perforation marked the site where the membrane would eventually peel off in layers to yield the one to two thin cell layers of the healed membrane (Fig. 3K,L). There was evidence of cornification at the junction between the healthy OEL and the ‘peeling-off’ of terminally differentiated cells (Fig. S2), suggesting this process to be the mechanism by which the membrane removes unwanted cells and returns to its original thin structure.

### Mesenchymal cells use the epithelial scaffold to cover the wound prior to invasion of the vasculature

**Fig. 4.**
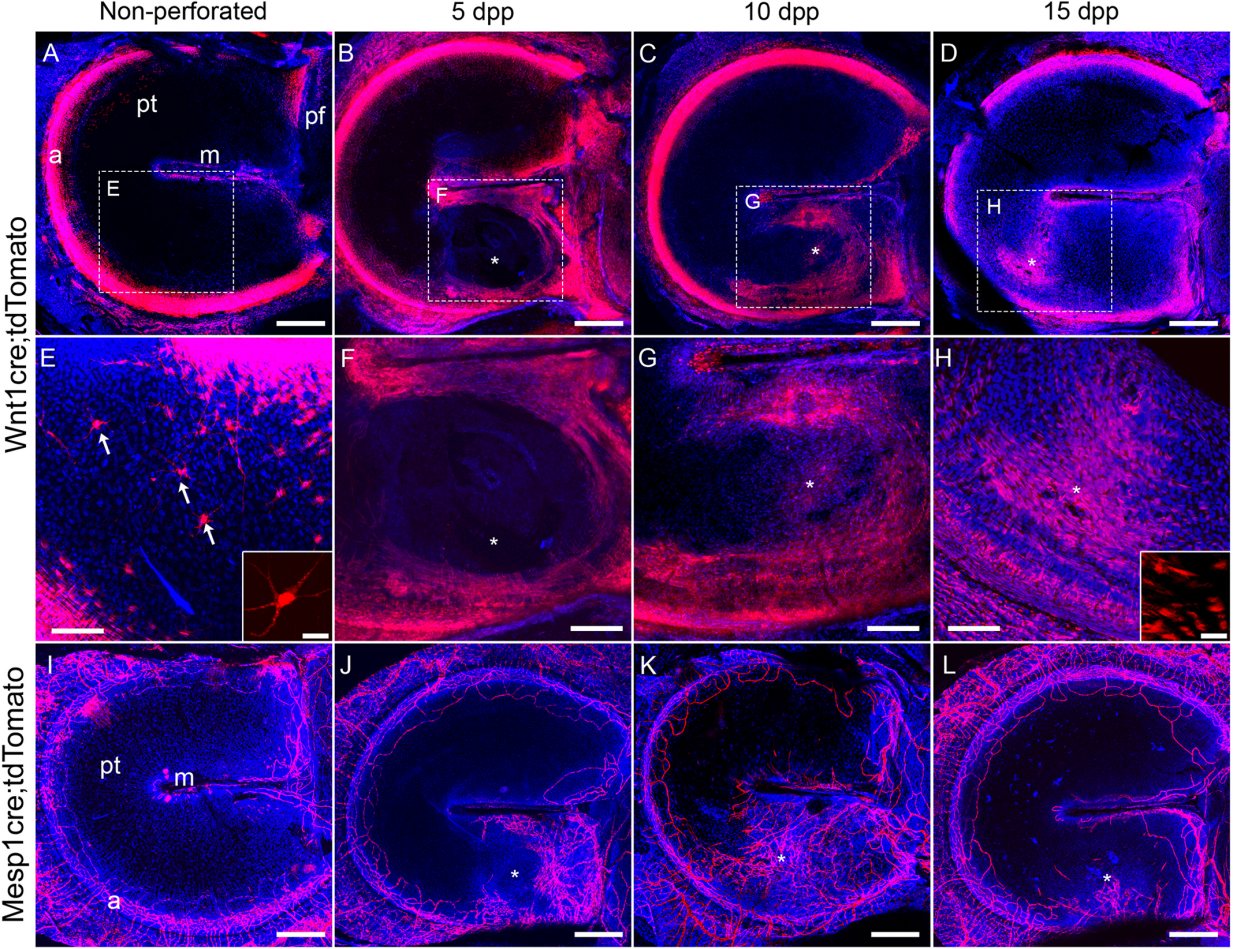
**Temporal dynamics of healing of the neural crest-derived tympanic membrane layer lamina propria and of mesoderm-derived blood vessels.** (A-H) Whole-mount fluorescence staining for RFP (red) in *Wnt1cre;tdTomato* reporter mice, labeling the neural crest-derived cells of the lamina propria. (I-L) Whole-mount fluorescence staining for RFP (red) in the *Mesp1cre;tdTomato* reporter mice labeling blood vessels. Nuclei are stained with DAPI (blue). Shown are non-perforated TMs, and TMs at 5, 10 or 15 days post perforation (dpp) as indicated. Boxed areas in A-D are shown magnified in E-F, respectively. Bottom right insets in E and H show representative cells at high magnification. Notice that brightness was increased in E to visualize the sparce individual cells (arrows) are not obvious in A. *n*=3 for each timepoint, *Wnt1cre;tdTomato* and *Mesp1cre;tdTomato.* Scale bars: 200 µm (A-D, I-L), 100 µm (E-H), 25 µm (insets in E and H). a, annulus tympanicus; m, manubrium of the malleus; pf, pars flaccida; pt, pars tensa; asterisks (*) indicate the perforation site.

To identify the response of the middle layer during healing, eardrums of *Wnt1cre;tdTomato* reporter mice (see Materials and Methods) were used (Fig. 4A-H). During homeostasis, neural crest-derived cells were located around the annulus and the manubrium, with some sparse and isolated mesenchymal cells in the PT (Fig. 4A,E, arrows). The sparse nature of the neural crest cells within the main body of the TM, highlights how spaced out the cells are in this thin layer, which is dominated by extracellular matrix (see Fig. 2K). At 5 dpp, an increase of cells staining positive for the red fluorescent protein tandem dimer Tomato (tdTomato), suggestive of a thickening of the middle layer, was evident around the manubrium and annulus on the side of the perforation (Fig. 4B). At this stage, cells could be observed covering the original perforation site, but no tdTomato-positive neural crest lineage cells were observed immediately around the closed wound (Fig. 4F, asterix). By 10 dpp (Fig. 4C,G) tdTomato-positive cells had migrated into the site of the healing perforation, with a further increase in the number of tdTomato-positive cells by 15 dpp (Fig. 4D,H). Interestingly, in the unperforated TM, mesenchymal cells had a clear star-shaped morphology with long projections (Fig. 4E arrows, inset), while at 15 dpp the mesenchyme cells within the healing wound were more compact and elongated (Fig. 4H, inset).

**Fig. 5.**
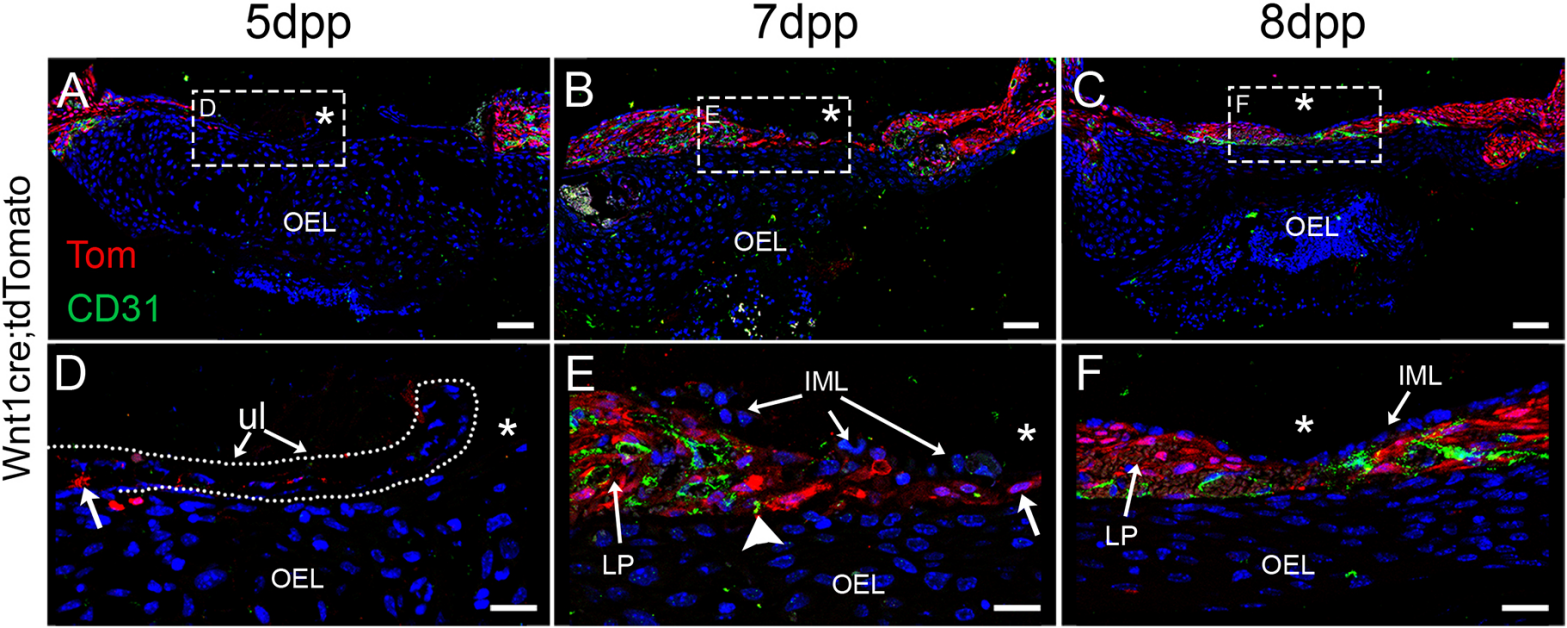
**Sequential involvement of neural crest-derived lamina propria and vasculature following the epithelial scaffolding.** (A-F) Immunofluorescence images of transversal sections at the perforation site in the *Wnt1cre;tdTomato* reporter mouse stained for RFP (Tom, red) and CD31 (green) at 5, 7 or 8 days post perforation (dpp) as indicated. Nuclei were stained with DAPI (blue). Boxed areas in A-C are shown magnified in D-F, respectively. Asterisks (*) indicate the perforation site. Unlabelled arrows in D and E indicate the advance front of tdTomato-positive cells of the lamina propria, other arrows indicate layers as labelled. Arrowhead in E indicates the advance front of CD31-positive cells of the blood vessels. IML, inner mucosal layer; LP, lamina propria; OEL, outer ectodermal layer; ul, unretracted layers. Scale bars: 50 µm (A-C), 20 µm (D-F). *n*=2 mice per timepoint.

The middle layer (i.e. the lamina propria) also contains the vasculature of the TM (Mozaffari et al., 2020). The timing of the invasion of the vasculature was followed in *Mesp1cre;tdTomato* mice, where *Mesp1* drives *tdTomato* expression in mesodermal derivatives, including endothelial cells (Asrar and Tucker, 2022). In unperforated TMs, blood vessels were observed around the annulus and the manubrium, while the centre of the PT was devoid of vasculature (Fig. 4I). At 5 dpp, a large change was observed in the blood vessel network, with an increase of mesoderm-derived cells around the annulus and manubrium on the side of the perforation, mimicking the changes in the mesenchymal layer (Fig. 4J). The centre of the wound, however, remained devoid of any vasculature until 10 dpp (Fig. 4J,K). By 15 dpp the blood vessels had started to disappear and/or retract from the newly healed membrane, with a similar location to control conditions (Fig. 4L). The lamina propria and its vasculature, therefore, invade the wound site after the ectodermal derived layer (Fig. 4). To further investigate the interactions between neural crest derived lamina propria and endothelial cells during the healing process, we analysed the spatial distribution of these cell types relative to the perforation site (Fig. 5). For this, CD31 was used to label endothelial cells (Asrar and Tucker, 2022) and *Wnt1cre;tdTomato* to label the mesenchymal cells of the lamina propria. At 5 dpp (Fig. 5A), a few tdTomato-positive cells (see arrow) were observed near the site of perforation (asterisk), while no CD31-positive cells were detected in this area (Fig. 5A,D). By 7 dpp (Fig. 5B), a noticeable thickening of the lamina propria was evident close to the perforation (asterisk), with individual tdTomato-positive cells at the advancing edge (arrow) bridging the wound. In contrast, the CD31-positive cells were situated at a distance (arrowhead) from the site of perforation (asterisk) within the already thickened lamina propria (Fig. 5E). At 8 dpp (Fig. 5C), both cell types had bridged the perforation (asterisk), although the central region had not reached its full thickness (Fig. 5F). These observations indicate that the initial response involved the migration of a single cell layer of neural crest cells to cover the perforation, followed by subsequent thickening and advancement of the vasculature.

### The mucosal layer seals the perforation wound simultaneously with the lamina propria

**Fig. 6.**
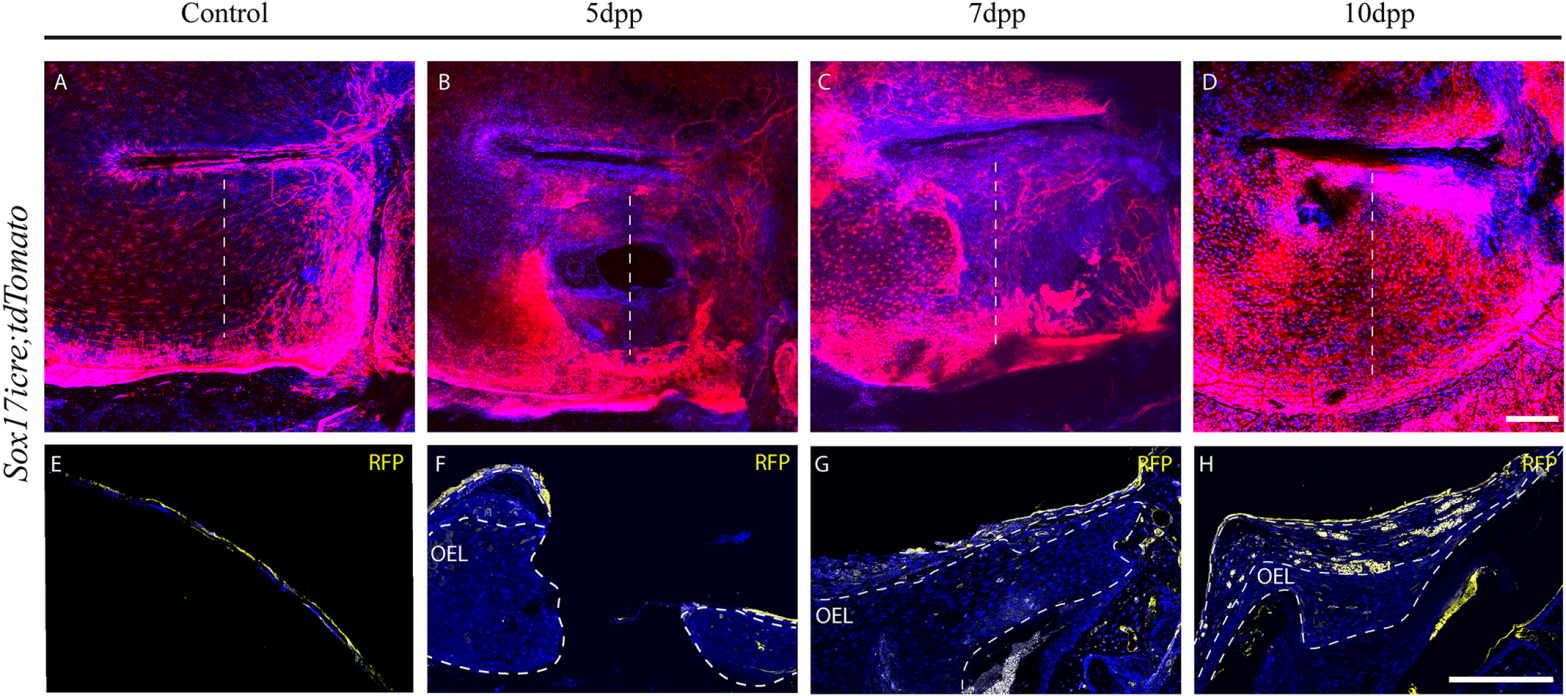
**The inner mucosal layer repairs in tandem with the lamina propria.** (A-D) Whole-mount immunofluorescence images of the RFP fluorescence labelling the inner mucosal layer (IML) in a *Sox17icre;tdTomato* reporter mouse. Dashed vertical lines indicate the level of the respective sections. (E-H) Transversal sections through the eardrum in the *Sox17icre;tdTomato* mouse. Immunofluorescence of tdTomato (RFP, yellow) labelling the IML. (A,E) Unperforated eardrum (Control). (B,F) 5 days post perforation (5 dpp). (C,G) 7 dpp. (D,H) 10 dpp. Nuclei were stained with DAPI (blue). Areas surrounded by dashed lines to denote the different layers of the TM. Scale bars: 200 µm. (D,H). *n*=2 mice per timepoint. Immunofluorescence assays were repeated three times on different specimens. OEL, outer ectodermal layer.

Finally, the response of the inner endoderm layer was followed by using the *Sox17icre;tdTomato* mouse line, in which endodermal derivatives as well as the vasculature is labelled (Engert et al., 2009). In unperforated TMs, endoderm-derived cells were observed clearly marking the IML (Fig. 6A,E). At 5 dpp there was no evidence of the IML creating an early bridge (Fig. 6B,F), as has previously been suggested by Johnson and Hawke (1986). A layer of mucosal cells was evident up against the wound margin (Fig. 6B,F). By 7 dpp, the IML was observed as a continuous layer bridging the original perforation and overlying the other layers on the inside of the drum (Fig. 6C,G). This thin layer was retained at 10 dpp (Fig. 6D,H). The IML, therefore, moved in a similar timeframe to the underlying lamina propria, bridging the site of perforation after creating an outer epithelial scaffold.

### Proliferation of the epithelial layer occurs at specific sites while proliferation of the middle and inner layers occurs across the entire area

**Fig. 7.**
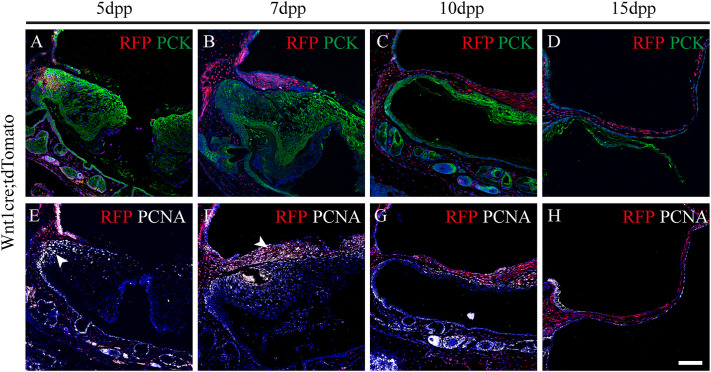
**Differential proliferation patterns between the three TM layers during healing.** (A-D) Immunofluorescence images of transversal sections of tympanic membranes (TMs) from *Wnt117cre;tdTomato* mice stained for red fluorescent protein (RFP, red) and the epithelial marker pan-cytokeratin (PCK, green) at 5 days (A,E), 7 days (B,F), 10 days (C,G) and 15 days (D,H) post perforation (dpp). (E-H) Immunofluorescence images of TMs as shown in A-D, stained for RFP (red) and the proliferation marker PCNA (white) in consecutive sections as A-D. Nuclei were stained with DAPI (blue). Arrowheads indicate proliferating regions. Scale bar: 100 µm. *n*=3 (5 dpp and 15 dpp), *n*=2 (10 dpp).

To understand the cellular behaviour of the TM during healing and correlate it to changes in thickness observed at different time points (see Fig. 1I-K), the proliferation pattern of the different layers was analysed by staining for proliferating cell nuclear antigen (PCNA). In sections of perforated TMs obtained from *Wnt1cre;tdTomato* mice, co-staining for pan-cytokeratin confirmed that the initial bridge was created by an epithelial spur prior to migration of the middle layer (labelled with tdTomato) and the IML (nuclei stained with DAPI) on the opposite site of the pan-cytokeratin-positive layer (Fig. 7A). Following the proliferative cells by using PCNA staining at 5 dpp, it was observed that the epithelial proliferation occurred at the annular and manubrial sides of the perforation (Fig. 7E, arrowhead), rather than at the ‘leading edge’, which remained free from proliferation (Fig. 7E). In contrast, the mesenchymal cells next to the annulus were non-proliferative at this time point (Fig. 7E).

At 7 dpp, a thicker mesenchymal layer was evident moving over the epithelial scaffold to reach the wound (Fig. 7B). The mesenchyme was highly proliferative at this stage – as shown by tdTomato and PCNA co-staining (Fig. 7F) – but, unlike the epithelial layer, the entire mesenchyme proliferated rather than this process occurring only near the margins. At this stage, the mucosal layer appeared to move with the underlying neural crest. Proliferative cells were observed along the IML (Fig. 7F), despite the slight increase in thickness of this layer throughout the healing process (Fig. 7B-D).

By 10 dpp, the mesenchymal layer had traversed the entire perforation and continued to proliferate (Fig. 7C,G), while proliferation of the epithelial layer was largely restricted to the basal layer (Fig. 7G). By 15 dpp, proliferation levels had decreased, with epithelial proliferation restricted to basal cells and very limited proliferation evident in the mesenchyme or endodermal layers of the TM (Fig. 7D,H).

## DISCUSSION

**Fig. 8.**
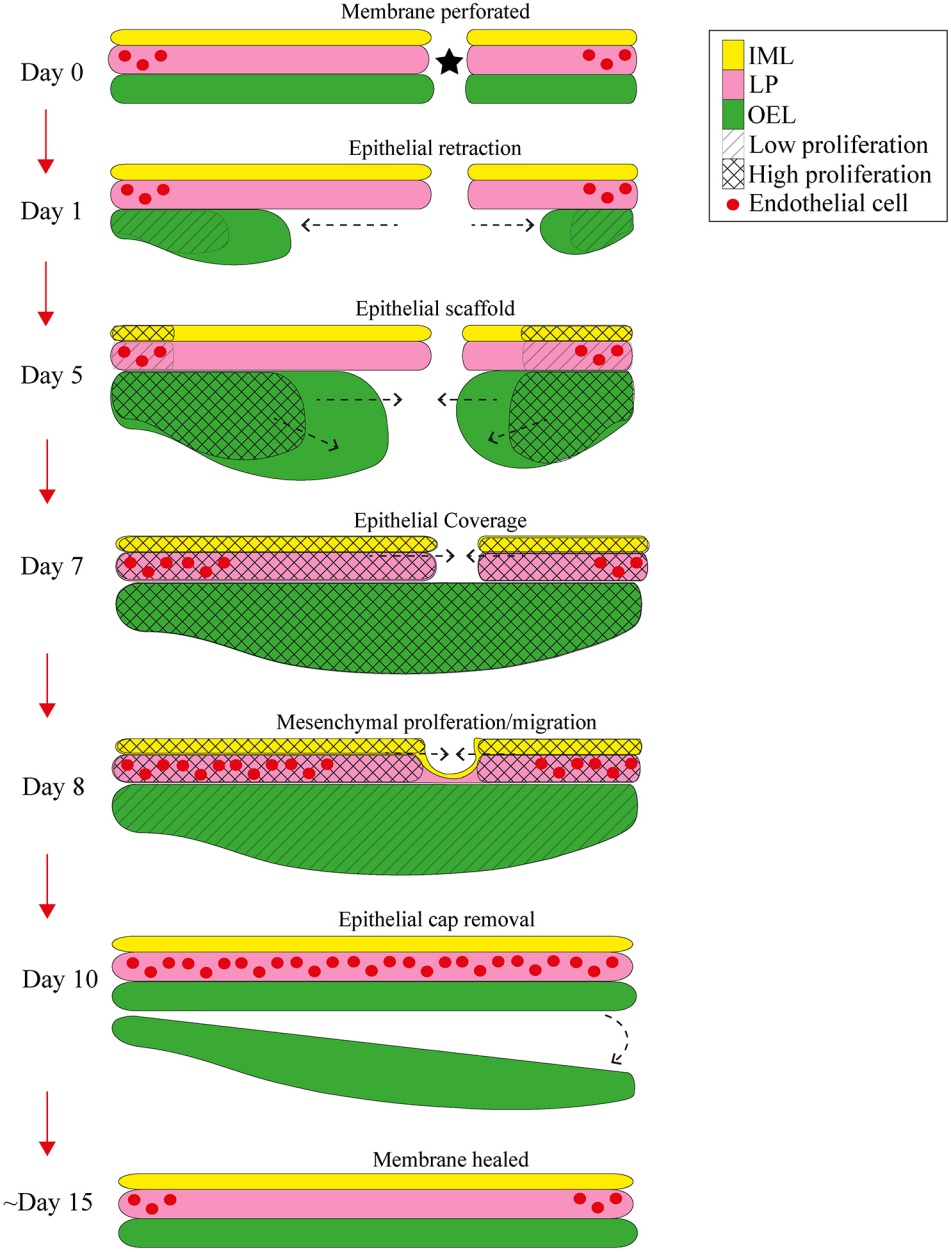
**Schematic of the healing process after acute tympanic membrane perforation**. Shown is the healing process of the three layers of the tympanic membrane (TM) after perforation. After perforation (Day 0), the outer epithelial layer (OEL) retracts with the lamina propria (LP) and inner mucosal layers (IMLs) remaining. The OEL proliferates at the junction of the annulus and manubrial areas, to form a scaffold between days 5 and 7. After scaffold formation, the LP and IML proliferate and cover the wound (days 7-10), utilising the epithelial scaffold formed before. Following the LP are endothelial cells, which migrate just behind the LP (days 7-10) to form the vasculature of the TM by day 10. The mass of tissue formed from OEL scaffold formation is lost by day 10, and the tissue thins down during the remodelling phase (day 15). Linear hatched regions denote regions of low proliferation. Crosshatched regions denote regions of high proliferation. Asterisk indicates site of perforation. Arrows indicate direction of movement of layers.

In this study the contribution of distinct TM layers was assessed during healing after TM perforation. By using immunofluorescence and genetic reporter mouse lines, rather than relying on histological approaches, it was possible to characterise the contribution of the layers of the TM accurately and reproducibly. After perforation, the outer epithelium was shown to retract by 2 dpp, with the lamina propria and IMLs remaining. The OEL proliferated at the junction between the annulus and manubrium area, which pushed the cells over the perforation to form a scaffold between 5 and 7 dpp. Variations in timing of closure of the gap might depend on subtle differences in position or initial size of the perforation, although variation was reduced for both by visualising the perforation site using an endoscope during surgery. After expansion of the OEL, the mesenchymal and inner layers then utilised this scaffold to cover the wound, followed by invasion of the vasculature. Closure of the wound site was followed by a remodelling step, whereby the outer epithelial scab was lost, and the tissue thinned down to its original functional size. Fig. 8 highlights the mechanisms of action during the healing process in response to TM perforation.

Our results highlight that, after perforation, significant retraction of only the OEL took place. This retraction was not immediate, with no evidence at 3 h post perforation, suggesting it did not involve a reflex contraction. However, it is likely that the retraction was triggered by changes in tension in the usually tight membrane. While there are examples of epithelial retraction involved in healing, e.g. during healing of the cornea (Onochie et al., 2019), it has not been described at the scale observed here. Whether retraction of the epithelium is an integral part of the healing process is unclear but it did only affect the OEL – the first tissue to respond to injury, with localized proliferation to push cells over the hole. The OEL showed heightened levels of phalloidin compared to those in non-retracted layers, suggesting that actin helps the non-proliferating epithelial cells at the retraction edge to contract over the wound by using a purse-string mechanisms (Martin and Lewis, 1992).

While previous studies narrowed down areas of proliferation to the side of the TM where the perforation has been made (Chari et al., 2019), we further show that the proliferative zones were restricted (during epithelial scaffold formation) to the annulus and manubrium either side of the perforation, with newborn cells pushed away from these regions towards the wound site. Initially, the bridging epithelial cells were KRT5 positive, agreeing with previous reports (Chari et al., 2019), but later differentiated into KRT10- and loricrin-positive cells, showing their ability to mature within days. The ability of the enlarged epithelial layer to ‘thin down’ was explained by loricrin-positive cells peeling off in layers into the ear canal, from 10 dpp to the end of the remodelling phase. During this process, cells to be shed appeared to flatten and to lose their nuclei, which is characteristic of cornification, a non-apoptotic form of programmed cell death (Eckhart et al., 2013) (Fig. S2).

The mesenchymal reaction began towards the end of or after the epithelial scaffold formation at ∼5 dpp. In contrast to the epithelial layer, proliferative cells were observed throughout the re-forming mesenchyme from 7 dpp, suggesting the tissue does not have a ‘pushing mechanism’ as such because, unlike the epithelia, the mesenchymal and inner layers can use the epithelial scaffold to travel across the air-borne middle ear. The mucosal layer contributed to the wound closure in tandem with the mesenchymal layer, although the observed increase in thickness was earlier (5 dpp) than that of the middle layer (7-10 dpp) (Fig. 1J,K). Shedding of tissue during remodelling of the scar, therefore, only occurred substantially on the external ear side of the membrane. In our analysis, the middle layer started to thin down soon after 10 dpp, indicating that the remodelling phase had begun (Santa Maria et al., 2011). How the mesenchymal layer ‘thins’ after its 'proliferative ‘event’ is of interest and worth further investigation. As the mesenchymal layer is sandwiched between the outer and inner layers, cells do not have the freedom to ‘peel off’ into the environment. It is possible that apoptosis has a role, although apoptotic bodies were not evident after staining with DAPI (data not shown), or phagocytosis by macrophages, which eliminate diseased and damaged tissue in other systems (Hirayama et al., 2017). Vascularisation is important for bringing in macrophages and other immune cells, while providing a supply of nutrients during later stages of repair. Increases in the vasculature network occurred after bridging of the wound by the neural crest-derived mesenchyme, with a reduction after 10 dpp and complete disappearance from the wound site once fully healed. Whether the vessels migrated out or were removed through apoptosis during the remodelling phase is an interesting avenue to follow.

Overall, our findings are important because tympanic membrane perforation and, even more, chronic tympanic membrane perforation has a very high incidence worldwide. Current options to treat chronic perforations are limited and surgery focussed. Moreover, although all perforations healed in our mouse model, our research suggests several mechanisms that can disrupt the healing process. The annulus tympanicus and manubrium of the malleus were heavily involved in the repair process, providing cells for closure, which agrees with the finding that these regions contain putative stem cell populations (Tucker et al., 2018). In contrast to the main body of the membrane, perforations within these regions would, therefore, be predicted to heal less well if the stem cell compartment was damaged. In human patients, bilateral repair of holes has been documented (Yamashita, 1985). This could be explained by defects in migration of the neural crest-derived layer that moves in after the outer epithelium. Such migration defects could be a direct issue with the neural crest or could be caused by the epithelial layers as excess proliferation could physically obstruct movement of the other layers. Finally, it would be interesting to see how infection, such as otitis media, affects the different layers during healing of a perforation, which should be possible by crossing krt5creER, *Wnt1cre* or *Sox17icre* reporter mice to otitis media mouse models (Fons et al., 2022). By understanding the contribution of each cell layer in the healing process it may be possible to generate targeted therapeutics for chronic perforations in the future.

## MATERIALS AND METHODS

### Animals

All mice were kept in the Biological Services Unit at King's College London. All animal husbandry and procedures were carried out in accordance with King's College ethical guidelines and UK Home Office guidelines. *Wnt1Cre*- (Danielian et al., 1998), *Mesp1Cre*- (Saga et al., 1999), *Sox17-2A-iCre*- (Engert et al., 2009) expressing males and *Rosa26;Tomato*-expressing females (*tdTomato* [*Gt(ROSA)26 Sor tm14(CAG-tdTomato) Hze*/J]; The Jackson Laboratory) were bred to create the reporter mouse lines *Wnt1cre;tdTomato*, *Mesp1cre;tdTomato* and *Sox17icre;tdTomato*. Reporter lines were bred on the C57BL/6 background. Control mice in Fig. 1 were on the CD1 background. No difference was observed between lines regarding rates of healing. *n* numbers in figure legends correspond to numbers of mice. In most experiments, one eardrum was perforated with the contralateral eardrum of the same animal used as a control.

### Perforation of the tympanic membrane

Eardrum, i.e. tympanic membrane (TM), perforation was performed in 6-8 weeks old wild-type CD1 (*n*=20), *Wnt1cre;tdTomato* (*n*=13), *Mesp1cre;tdTomato* (*n*=12) and *Sox17icre;tdTomato* (*n*=8) adult mice. Transgenic mice were on a mixed CD-1 and C57BL/6 background. Both male and female mice were used for experimentation. All eardrums healed by 15 days with no difference shown for sex. Perforations were created using a 27-g syringe and 1-mm endoscope camera for visualisation (Fig. 1B) (RVA synergies) in animals anaesthetised with a cocktail of ketamine and medetomidine at 75 mg/kg. After surgery, anaesthesia was reversed using atipamezole and buprenorphine. At specific time points (i.e. 3 h, and 2, 5, 7, 10 or 15 dpp) mice were killed using approved schedule 1 culling methods (Scientific Procedures. Act 1986), and eardrums, attached to the tympanic ring, were dissected under a Leica MZFLIII dissection scope. Analysis was not conducted longitudinally in the same animal. Dissected tissue was fixed in 4% paraformaldehyde for 1 h and DAPI stained prior to whole-mount imaging. All animal surgery was approved by the UK Home office with licences in place.

### Cell staining using CM-DiI

Perforations were conducted as described above but the needle used to perform the perforation was dipped in CM-DiI dye (Invitrogen, #C7000) prior to perforation. Using this method, cells at the site of perforation were stained with DiI and could be followed during the healing process to observe their fate.

### Histology and immunohistochemistry

For paraffin embedded sectioning, samples were decalcified in 0.5 M EDTA (pH 9) for 16 h at room temperature (RT), dehydrated by using PBS and an ethanol dilution series (30%, 50%, 70%, 90%, 100%) for 30 min each, cleared using xylene and embedded in paraffin wax. 6 μm sections were cut using a Leica microtome RM2245 and mounted onto super frost slides (Fisher Scientific, #11976299). Immunofluorescence was performed as follows: the paraffin slides were initially dewaxed in xylene (3×10 mins) and rehydrated using an ethanol dilution series (100%, 90%, 70%, 50%, 30%) and PBS for 2 min each. Antigen retrieval was achieved using 0.01 M citrate buffer (pH 6) in a water bath at 95°C. After antigen retrieval slides were washed with PBS and blocked using blocking buffer (10% FBS, 1% BSA and 0.0125% Tween-20 in PBS for 1-2 h). Primary antibodies were applied overnight at 4°C (see supplementary material Table S1) and washed out (4× for 5 min) with PBT [phosphate-buffered saline (PBS) pH 7.5 with 0.0125% Tween 20] before incubation with secondary antibodies (including DAPI) made up in blocking solution (see supplementary material Table S1). Finally, slides were washed in PBT (4×5 min), mounted using Fluoroshield (Sigma, # F6182) and left to dry overnight before imaging. TM sections tend to detach from the slide. To avoid this, the antigen retrieval buffer was not pre-heated and a small percentage of detergent (0.0125% instead of the standard 0.1%) was used in the buffers.

This protocol was followed for all immunofluorescence experiments apart from that for type II collagen, which requires a pre-antigen retrieval step whereby slides were treated with 3% hydrogen peroxide for 30 mins at RT. Slides were also processed by using a trichrome stain with Sirius Red, Ehrlich's haematoxylin and Alcian Blue under standard protocols. Immunofluorescence experiments were repeated on 2-3 TM samples for each timepoint analysed.

### Imaging

Whole eardrums on fluorodishes (World Precision Instruments, #FD35-100) or slides stained with fluorescent antibodies were imaged on a Leica TCS SP5 confocal microscope by using LAS AF software. Trichrome stained slides were imaged on the Nikon Eclipse 80i and brightfield images were taken using the Leica Flexera A5. Images were processed and quantified using ImageJ (version 1.0), figures (including schematics) were made using Adobe Illustrator (2021) or Adobe Photoshop (2021).

### Quantification and statistics

Quantification of membrane thickness and perforation areas were carried out using ImageJ (version 1.0). Quantification measurements for membrane thickness were taken as shown in Fig. S1. Statistical analysis of TM perforation areas was carried out using a one-way ANOVA with Tukey's post hoc test to compare three conditions. Statistical analyses were performed using GraphPad Prism9. *n* values for statistical tests were defined using power calculations.

## Supplementary Material

10.1242/dmm.050466_sup1 Supplementary information
