# Supplementary material for: Tracking cell layer contribution during repair of the tympanic membrane
Source: Dis Model Mech. 2024 Mar 28;17(3):dmm050466. doi: 10.1242/dmm.050466 (PMC10985735; doi:10.1242/dmm.050466)
Supplement: Supplementary information [file dmm-17-050466-s1.pdf]

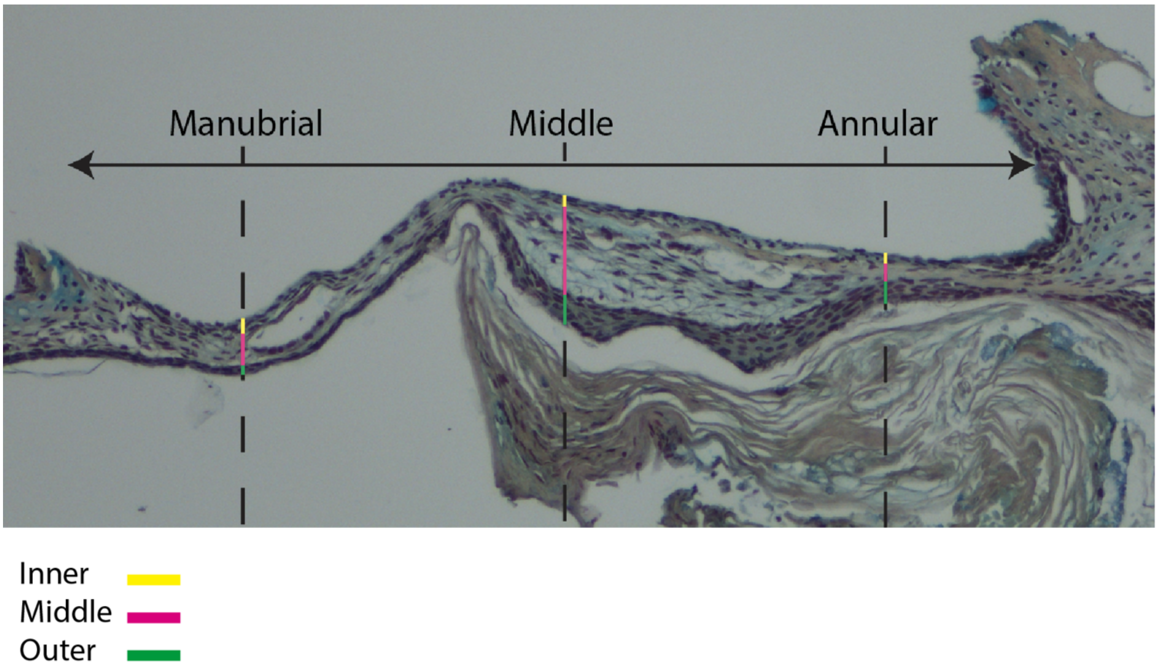

Fig. S1. Representative image of the protocol for measuring thickness of the tympanic membrane.

Image to show where the measurements were taken when quantifying thickness of the TM was at various timepoints for the graphs in Figure 1. A measurement of half the TM was taken (arrow-to-arrow), and this number was split into thirds. At each third a measurement was taken (represented by the coloured lines) to achieve measurements at manubrial, middle and annular regions of the TM. Each measurement was taken for each of the layers of the TM, identifiable via trichrome stain. n=4/timepoint.

Disease Models & Mechanisms • Supplementary information

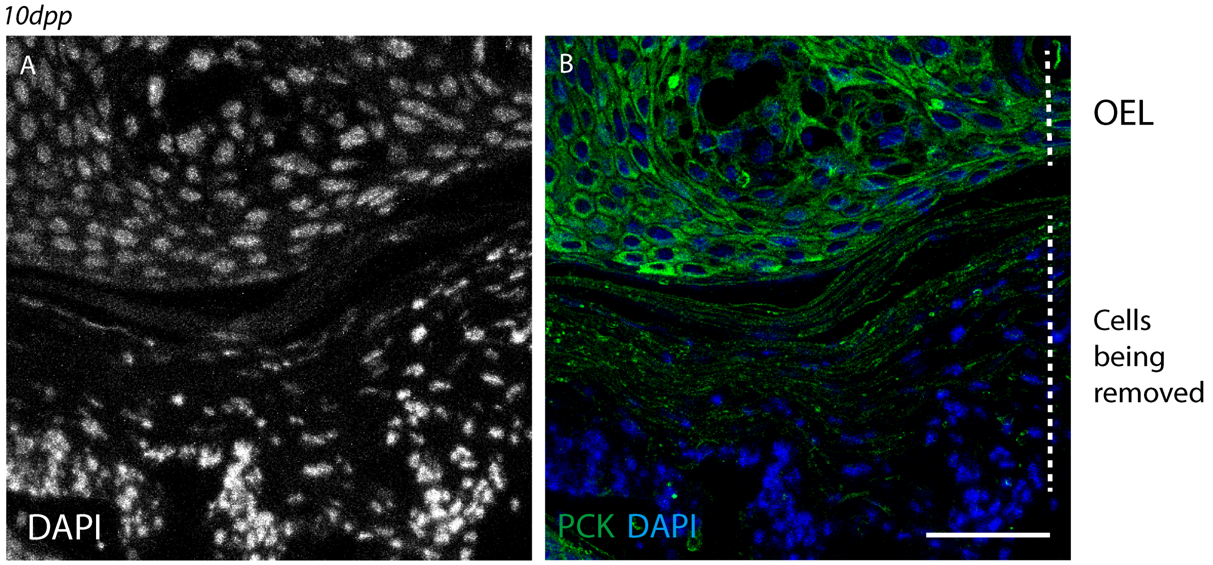

Fig. S2. The outer epithelial layer undergoes cornification at 10dpp

Healing TM at 10dpp stained for DAPI (A) to observe nuclei shape, and Pancytokeratin and DAPI (B) to reveal structural differences between cells in the outer epithelial layer and cells being removed via cornification. n=3 10dpp samples, immunofluorescence assay repeated 3 times on different specimens. Scalebar = 50µm.

Table S1. Table of Antibodies

Antibody	Species	Manufacturer	Catalogue Number	Immunofluorescence Dilution
Phalloidin	Na	Invitrogen	A12379	1:1000
Pancytokeratin	Rabbit	Abcam	Ab217916	1:200
RFP	Goat	Sicgen	Ab1140-100	1:200
PCNA	Rabbit	Abcam	Ab19166	1:200
Collagen (II)	Mouse	DSHB	II-II6B3	1:50
Keratin 5	Rabbit	Covance	PRB-160P	1:300
Keratin 10	Mouse	Abcam	Ab76318	1:300
Loricrin	Rabbit	Biolegend	PRB-145P	1:200
CD31	Rabbit	Abcam	Ab182981	1:200
Anti-goat Secondary Antibody, Alexa Fluor 488	Donkey	Invitrogen	A-11055	1:500
Anti-Rabbit Secondary Antibody, Alexa Fluor 488	Goat	Invitrogen	A-11008	1:500
Anti-Mouse Secondary Antibody, Alexa Fluor 488	Goat	Invitrogen	A-11001	1:500
Anti-Rabbit Secondary Antibody, Alexa Fluor 568	Donkey	Invitrogen	A10042	1:500
Anti-Mouse Secondary Antibody, Alexa Fluor 568	Donkey	Invitrogen	**A10037**	1:500
Hoechst	Na	Thermofisher Scientific	62249	1:1000

Disease Models & Mechanisms • Supplementary information
